# Calcium and vitamin D intakes in children: a randomized controlled trial

**DOI:** 10.1186/1471-2431-13-86

**Published:** 2013-05-23

**Authors:** Linda Cosenza, Vincenza Pezzella, Rita Nocerino, Margherita Di Costanzo, Anna Coruzzo, Annalisa Passariello, Ludovica Leone, Marcella Savoia, Antonio Del Puente, Antonella Esposito, Gianluca Terrin, Roberto Berni Canani

**Affiliations:** 1 Department of Pediatrics, University of Naples “Federico II”, Naples, Italy; 2 European Laboratory for the Investigation of Food Induced Diseases, University of Naples “Federico II”, Naples, Italy; 3 Department of Biochemistry and Medical Biotechnologies, University of Naples “Federico II”, Naples, Italy; 4 Department of Clinical and Experimental Medicine, University of Naples “Federico II”, Naples, Italy; 5 Department of Women Heath and Perinatal Medicine, University of Rome “La Sapienza”, Rome, Italy

**Keywords:** 25-hydroxyvitamin D, Dietary counseling, Pediatrics, Ca^2+^ intake, VitD intake, Bone metabolism, Nutritional intervention, Vitamin D supplement, Vitamin D deficiency

## Abstract

**Background:**

Calcium (Ca^2+^) and vitamin D (VitD) play an important role in child health. We evaluated the daily intake of Ca^2+^ and VitD in healthy children. Moreover, we demonstrate the efficacy of Ca^2+^ and VitD supplementation.

**Methods:**

Daily Ca^2 +^ and VitD intake was evaluated in consecutive healthy children through a validated questionnaire. Subjects with <70% of dietary reference intakes (DRIs) of Ca^2+^ and VitD were invited to participate in a prospective randomized trial with 2 groups of nutritional intervention: Group 1, dietary counseling aiming to optimize daily Ca^2+^ and VitD intake plus administration of a commercially available Ca^2 +^ and VitD supplementation product; Group 2, dietary counseling alone. At the enrollment (T0) and after 4 months (T1) serum 25(OH) Vitamin D levels were assessed.

**Results:**

We evaluated 150 healthy children (male 50%, mean age 10 years); at baseline a low VitD intake was observed in all subjects (median 0.79 μg/die, IQR 1.78; range 0.01-5.02); this condition was associated with Ca^2+^ intake <70% of the DRIs in 82 subjects (55%). At baseline serum 25(OH)D levels were low (<30 ng/ml) in all study subjects and after 4 months of nutritional intervention, a normalization of serum 25(OH)D levels (≥30 ng/ml) was observed in all children in Group 1 and in only one subject in Group 2 [Group 1: T1 33.8 ng/ml (IQR 2.5) vs Group 2: T1 24.5 ng/ml (IQR 5.2), p <0.001].

**Conclusions:**

Adequate Ca^2+^ and VitD intakes are difficult to obtain through dietary counseling alone in pediatric subjects. Oral supplementation with of Ca^2+^ and VitD is a reliable strategy to prevent this condition.

**Trial registration:**

The study was registered in Clinical Trials Protocol Registration System (ID number: NCT01638494).

## Background

Adequate dietary intake of calcium (Ca^2+^) and vitamin D (VitD) in children is important to guarantee normal bone mineralization and for rickets prevention. Bone is the main store of Ca^2+^, it is a reserve for the homeostasis of serum Ca^2+^concentration, which varies within narrow limits around 2.5 mmol/L (10 mg/dL). Apart from its main structural role, Ca^2+^ is also important in coagulation cascade, in neuromuscular excitability and contraction, in enzyme hormones and growth factors activity, in secretion of hormones and in cell growth and differentiation [1]. Dietary Recommended Intake (DRI) for Ca^2+^ is 700 mg/day for children aged 1–3 years, 1000 mg/day for children aged 4–8 years, and 1300 mg/day for children aged 9–10 year and adolescents [2].

The metabolism of Ca^2+^ closely depends on VitD, parathormone and calcitonin levels. Vitamin D stimulates intestinal absorption of Ca^2+^ and phosphorus, and regulates serum Ca^2+^ levels maintaining an adequate mineralization of the skeleton [3]. Apart for the fundamental role in bone and Ca^2+^ homeostasis, recent evidences suggest that VitD, through its active form, has pleiotropic roles in both innate and adaptive immunity. A correct intake of VitD has been implicated in the prevention of a number of conditions including autoimmunity, atopic disorders, certain forms of cancer, chronic hepatitis C, obesity and cardiovascular diseases [4-13]. According with these findings the American Academy of Pediatrics (AAP) changed the recommendations on VitD intake. The new recommended daily intake is 400 IU for infants, children and adolescents [2]. Unfortunately, despite this recommandation, studies have reported a high rate of VitD insufficiency in the pediatric age in Western countries [14,15]. These results have a multi-factorial origin, among these the insufficient intake related to dietary habits of children and adolescents is considered to be the most important factor. Moreover the efficacy and practicability of a dietary intervention aiming to normalize Ca^2+^ and VitD intake is still largely unknown. We investigated Ca^2 +^ and VitD intake and the efficacy and applicability of a nutritional intervention to aimed optimize Ca^2+^ and VitD intake in a population of healthy children.

## Methods

### Study design and population

The study protocol was approved by the Ethics Committee of the Medical School of the University of Naples "Federico II”. Otherwise healthy subjects (male and female, age range 3–17 years) consecutively observed as outpatients at our Department for routine clinical examination because vaccination program were considered eligible for the study. Exclusion criteria were: malnutrition (defined as a weight/height ratio <5° centile); presence of chronic systemic diseases (celiac disease, inflammatory bowel disease, food allergy, cystic fibrosis, malignancy, immunodeficiency, tuberculosis, genetic-metabolic disease, primitive bone disease, diabetes and endocrine disorder); use of systemic steroids in the previous 3 months. We excluded from the study even people who did not speak Italian properly and were not able to understand the Italian language and households non-resident in the Campania region. At the enrollment, information about the aim of the study were given to children’s parents and a written consent was collected from those who agreed to participate. The study was registered in Clinical Trials Protocol Registration System (ID number: NCT01638494).

### Intervention and data collection

At the enrollment, data regarding auxological parameters and general clinical conditions were assessed by 3 pediatricians unaware of the study aims. Then, all enrolled subjects were carefully evaluated by registered dieticians, with a wide experience in pediatric nutrition, assessing the dietary Ca^2+^ and VitD intake by a 3-day diary. The parents received written and oral instructions on how to keep the diary, recording everything that the child ate or drank and noting the quantities using household measures. Picture models of sizes of food that helped parents to estimate portion sizes were also provided. Further assumptions of Ca^2+^ and/or VitD or fortified foods were also registered. All data were recorded in a specific clinical chart and were analyzed using a specific software based on the Italian food composition tables (Winfood, Medimatica SRL, Martinsicuro, Teramo, Italy). All subjects with less than 70% of Ca^2+^ and VitD DRIs were invited to participate in the study. Using a computer generated list, these subjects were randomly allocated into one of 2 groups of nutritional intervention: Group 1, receiving dietary counseling to improve daily Ca^2+^ and VitD intake, plus administration of a commercially available Ca^2+^ and VitD supplementation product (Colecalcium Humana®, Milan, Italy; syrup: 10 ml/daily, containing 400 mg of Ca^2+^ and 400 IU of VitD); and Group 2, receiving dietary counseling alone. The dietary counseling, focused on feeding behaviors, selection pleasant food and children’s preferences was provided by dietitians unaware of the study aims. The dietitians gave information to the parents on how to improve the consumption of foods rich in Ca^2+^ and VitD according to DRIs, such as cod liver oil, salmon, mackerel, tuna fish, sardines, milk, cereal, egg, liver beef, cheese. No nutritional support products were prescribed in children enrolled in Group 2. In Group 1, Ca^2+^ and VitD supplementation product was given directly to the parents/tutors of the enrolled child. Each parent/tutor received eight bottles of the study product (150 ml/each) for the entire duration of the study. The families were asked to return the bottles at the end of the study period to check the adherence to the treatment. For all study subjects, data regarding demographic characteristics, auxologic parameters, outdoor sport activities, dietary Ca^2+^ and VitD intake, and adherence to the assigned treatment were collected in a specific clinical chart. At the enrolment (T0) and after 4 months of nutritional intervention (T1), all subjects underwent to a blood sampling (6 ml) to determine serum Ca^2+^ and 25 hydroxy vitamin D (25(OH)D) levels. For each study subject blood samples were obtained in the same season. Serum 25(OH)D levels were measured by a quantitative electrochemiluminescent method (Roche Elecsys assay, Roche Diagnostics SPA, Monza, Italy) and expressed as ng/ml. Serum Ca^2+^ concentration was measured using a spectrophotometer with a λ of 570 nm, and expressed as mg/dl. Serum Ca^2+^ results were compared with reference values obtained in our Center, evaluating more than 4000 healthy subjects of both sex and in the same age range of the study population.

### Statistical analysis

To demonstrate a difference of 25% in serum 25(OH)D concentration between the two study groups at T1, we estimated a sample size of 12 patients in each group in order to obtain a power of the study of 85% (type 1 error = 0.05; two-tailed test). This computation was based on the results of a preliminary trial (data on file). Our estimation of sample size allowed for a drop out of up to 20%.

Statistical analysis was performed by researchers blinded to the type of treatment received by the two groups of children using SPSS Version 16.0 for Windows (Chicago, Ill, USA). Results are reported as means and 95% confidence interval (CI) due to parametric distribution (Kolmogorov test). The Kolmogorov-Smirnov test was used to determine if variables were normally distributed. Non-parametric results are presented as median, IQR and range (min-max). For continuous variables, groups were compared using Mann–Whitney test and Wilcoxon test. For categorical variables, the χ2 test and Fisher’s exact test were used. The level of significance for all statistical tests was 2-sided, p< 0.05.

## Results

From December 2008 to February 2009, 184 consecutive children were considered eligible for the study. 34 children refused to participate, 150 subjects (male 50%, mean age 10 years, range 9.3-10.6, BMI 18.7, 17.9-19.5) filled the dietary questionnaire. All children were from the Campania region. Campania region is one of the most populated (5,834,056 residents), industrialized and sunny region of Italy, located in the south of the Country. The families well accepted the study procedures. A correctly filled questionnaire was provided by each parent/tutor. The median (IQR; range) daily intake of Ca^2+^ and VitD was 744.5 mg (427.2; 93–1679) and 0.79 μg, (1.8; 0.01-5.02) respectively.

**Figure 1:**
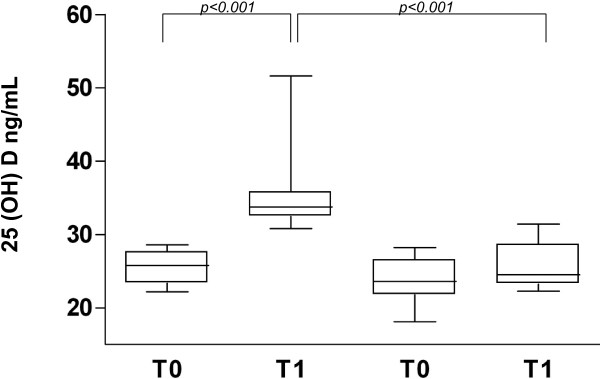
Serum 25(OH)D levels observed at baseline (T0) and after four months of nutritional interventions (T1) in the two study groups.

A total of 82 children (54.6%; male 50%, mean age 10.6 years, range 9.7-11.4, BMI 18.8, 17.9-19.8) showed a concomitant Ca^2+^ and VitD intake <70% of the DRIs. The first 24 subjects observed with these characteristics were invited to participate in the randomized trial. The families agreed to participate, but two subjects per group were lost at the follow-up and they were not considered for the final statistical analysis. The comparative study was performed from February to June 2009. The main demographic and clinical characteristics of the two study groups were similar (Table 1). A median of five hours per week of outdoor sport activities during afternoon was reported in both groups. At T0 the two groups showed similar Ca^2 +^ and VitD intakes (Tables 2 and 3). The supplementation with Ca^2+^ and VitD was well accepted in all study subject enrolled in Group 1. The adherence was optimal considering that all children consumed >90% of the daily dose during the entire study period. No adverse events were reported. At T1 children in Group 1 showed a simultaneous improvement of VitD and Ca^2+^ intake. At T1 children in Group 2 showed only a significant increase of Ca^2+^ intake, but not of VitD (Table 2). Serum Ca^2+^ levels were similar in the two groups at T0 and at T1. At baseline serum 25(OH)D levels were below the optimal value (≥30 ng/ml) [16] in both groups. After four months of nutritional intervention all subjects in Group 1 and only one child in Group 2 showed serum 25 (OH) D within normal value (Figure 1).

**Table 1:** Baseline main demographic and clinic characteristics of subjects enrolled in the randomized trial

	**Group 1**	**Group 2**
	***(dietary counseling plus Ca***^***2+***^***and Vitamin D supplementation)***	***(dietary counseling only)***
	**n=10**	**n=10**
**Age (years)**	13.3	11.4
	(9.9-16.6)	(8.5-14.1)
**Male**	4	6
**Weight (kg)**	49.0	44.6
	(37.1-60.9)	(29.9-59.3)
**Weight, percentile**	60	59
	(38–82)	(33–86)
**Height (cm)**	146	141
	(132–160)	(127–155)
**Height, Percentile**	40	43
	(17–63)	(15–71)
**BMI (kg/m**^**2**^**)**	22.1	21.3
	(18.9-25.2)	(17.7-24.9)
**BMI Percentile**	63	69
	(39–87)	(48–90)
**Outdoor sport activities (h/week)**	5	6
	(3–6)	(4–7)

**Table 2:** Calcium and Vitamin D daily dietary intake at baseline (T0) and after four months of nutritional intervention (T1) in the two study groups

	**Ca**^**2+**^**intake (mg/day)**			**VitD**_**3**_**intake (μg/day)**		
	**Group 1**	**Group 2**	***p***	**Group 1**	**Group 2**	***p***
**T0**	507	588	0.174	1.55	1.98	0.705
	(221; 93–728)	(353.5; 275–844)		(2.23; 0.03-4.3)	(3.03; 0.1-3.58)	
**T1°**	907.5*	925*	0.790	12.11**	2.40	<.001
	(217.75; 741–1591)	(401; 536–1201)		(3.40; 10.3-15.79)	(2.14; 0.43-5.38)	

° Calcium and vitamin D intake was calculated considering amount received by supplementation with the study product. *T0 vs T1 p<0.001; ** T0 vs T1 p=0.013.

Data are expressed as median (IQR; range).

**Table 3:** **Ca**^**2+ **^**and VitD**_**3 **_% **DRI at baseline (T0) and after four months of nutritional intervention (T1) in the two study groups**

	**% Ca**^**2+**^**DRI**			**% VitD**_**3**_**DRI**		
	**Group 1**	**Group 2**	***p***	**Group 1**	**Group 2**	***p***
**T0**	42.6	60.95	0.082	15.55	19.8	0.705
	(27.38; 7.75-61)	(24.22; 22.91-70.33)		(22.28; 0.3-43)	(30.33; 1–35.8)	
**T1**	75.62*	79.33**	0.722	121.15*	24	<.001
	(29.5; 61.75-198.87)	(31.15; 67–113.87)		(33.98; 103–157.9)	(21.4; 4.3-53.8)	

*T0 vs T1 p<0.001; ** T0 vs T1 p=0.008.

Data are expressed as median (IQR; range).

## Discussion

In the first part of the study we investigated Ca^2+^ and VitD intake in a cohort of healthy children afferent to our Department because of a vaccination program. This cohort was representative of a normal bright skin pediatric population living in an industrialized sunny area of South Italy where geographical alignment includes latitude of 42°-39° North. In all subjects we observed an inadequate dietary VitD intake, that was frequently associated (55% of cases) with inadequate Ca^2+^ intake.

The determination of serum 25(OH)D level in children enrolled in the second part of the study confirm an inadequate intake at baseline. The best indicator of VitD status is 25(OH)D concentration, which reflects absorption from the diet and synthesis by the skin [17]. Measuring concentration of 1,25-OH_2_-D instead of 25(OH)D for assessment of VitD status could be incorrect, because 1,25-OH_2_-D concentration could be normal or elevated in case of VitD deficiency as a result of secondary hyperparathyroidism [17]. Although there is no consensus on the optimal serum level of 25(OH)D, VitD insufficiency is commonly defined as 25(OH)D level between 21 to 29 ng/ml and VitD deficiency by 25(OH)D <20 ng/ml [16]. All study subjects enrolled in the second part of the study were below this limit. Our findings are consistent with those recently described in children living in Northern Italy, where a high prevalence of VitD deficiency (>50% of subjects) was observed [18].

The skin synthesis of VitD occurs during all around the year only at latitudes between the equator (latitude 0°) and about 37° North and 37° South. Outside this area the synthesis of VitD decreases progressively by moving toward the poles (latitude 90°) [16,19]. The Campania region is included in the so-called "VitD Winter zone". Thus during the winter season skin synthesis of VitD is generally inadequate. In fact, in winter season and at higher latitudes, the number of ultraviolet (UV) radiations reaching the Earth’s atmosphere decreases. As a consequence, small amounts of VitD are produced by the skin [20].

Until the 1990s the criteria for appropriate VitD intake was the absence of overt rickets or osteomalacia [17]. Our results support the concept that it could be wrong to assume that simply because children live in a sunny area, they need less VitD supplementation. Moreover, the Institute of Medicine (IOM) has recently revised its recommendations for Dietary reference intakes (DRIs) for VitD and calcium for US and Canadian populations. The IOM recommended daily intakes of VitD were increased from 200 IU to 400 IU in infants (0–12 months) and from 200 IU to 600 IU in children (1–18 years) [17]. These new DRIs support even more the results of our randomized trial. Despite the small sample size and problems deriving from a non-standardized nutritional guidelines, our results show that simple dietary counseling is insufficient to normalize VitD intake and 25(OH)D levels.

## Conclusions

Studies suggest that VitD insufficiency is a global public health problem across all life stages with serious short and long-term clinical consequences [4,21]. According to our findings, a dietary counseling alone is unable to obtain an adequate VitD intake that is necessary for body health and to reach optimal 25(OH)D serum levels. Unfortunately, the high prevalence of insufficient VitD dietary intake is largely unknown among clinicians and patients. We feel that long term strategies to address this silent disease should include public education and health policies for prevention, through food fortification and VitD supplementation.

## Abbreviations

Ca2+: Calcium; VitD: Vitamin D; DRIs: Dietary Reference Intakes; 25(OH)D: 25-hydroxyvitamin d; IOM: Institute of Medicine; CI: Confidence Interval; BMI: Body Mass Index; 1,25-OH2-D: 1,25-dihydroxycholecalciferol.

## Competing interests

The research was not funded by any Company. The commercially available calcium and Vitamin D supplementation product was a kind gift of Humana, Milan Italy. Humana staff did not participate in the protocol development, study oversight, regulatory reporting, monitoring the progress of the study, or manuscript preparation. No other conflicts of interested were reported. The authors acknowledge with gratitude the commitment of the Mother and Child Health Association (M.A.C.H.A.) to the research efforts.

## Authors’ contributions

LC, RBC, RN conceived the study, and participated in its design and coordination and wrote the first draft of the manuscript. LL, AC, MDC, VP, AP, MS, ADP, AE cared the patients, participated to acquisition of data, and revised the final version of the article. GT participated in the design of the study and performed the statistical analysis. All authors read and approved the final manuscript.

## Pre-publication history

The pre-publication history for this paper can be accessed here:

http://www.biomedcentral.com/1471-2431/13/86/prepub
